# Multiple-Clade H5N1 Influenza Split Vaccine Elicits Broad Cross Protection against Lethal Influenza Virus Challenge in Mice by Intranasal Vaccination

**DOI:** 10.1371/journal.pone.0030252

**Published:** 2012-01-18

**Authors:** Penghui Yang, Yueqiang Duan, Peirui Zhang, Zhiwei Li, Cheng Wang, Mei Dong, Chong Tang, Li Xing, Hongjing Gu, Zhongpeng Zhao, Xiufan Liu, Shaogeng Zhang, Xiliang Wang

**Affiliations:** 1 Beijing Institute of Microbiology and Epidemiology, State Key Laboratory of Pathogen and Biosecurity, Beijing, China; 2 Department of Hepatobiliary, 302 Military Hospital, Beijing, China; 3 Medical College of Soochow University, Suzhou, China; 4 Key Laboratory for Animal Infectious Diseases of Ministry of Agriculture, Yangzhou University, Yangzhou, China; The University of Hong Kong, China

## Abstract

**Background:**

The increase in recent outbreaks and unpredictable changes of highly pathogenic avian influenza (HPAI) H5N1 in birds and humans highlights the urgent need to develop a cross-protective H5N1 vaccine. We here report our development of a multiple-clade H5N1 influenza vaccine tested for immunogenicity and efficacy to confer cross-protection in an animal model.

**Methodology/Principal Findings:**

Mice received two doses of influenza split vaccine with oil-in-water emulsion adjuvant SP01 by intranasal administration separated by two weeks. Single vaccines (3 µg HA per dose) included rg-A/Vietnam/1203/2004(Clade 1), rg-A/Indonesia/05/2005(Clade 2.1), and rg-A/Anhui/1/2005(Clade 2.3.4). The trivalent vaccine contained 1 µg HA per dose of each single vaccine. Importantly, complete cross-protection was observed in mice immunized using trivalent vaccine with oil-in-water emulsion adjuvant SP01 that was subsequently challenged with the lethal A/OT/SZ/097/03 influenza strain (Clade 0), whereas only the survival rate was up to 60% in single A/Anhui/1/2005 vaccine group.

**Conclusion/Significance:**

Our findings demonstrated that the multiple-clade H5N1 influenza vaccine was able to elicit a cross-protective immune response to heterologous HPAI H5N1 virus, thus giving rise to a broadly cross-reactive vaccine to potential prevention use ahead of the strain-specific pandemic influenza vaccine in the event of an HPAI H5N1 influenza outbreak. Also, the multiple-clade adjuvanted vaccine could be useful in allowing timely initiation of vaccination against unknown pandemic virus.

## Introduction

Influenza infection continues to be a major threat to human health on several fronts. Influenza A (H5N1) viruses remain a major concern due to their evolution, genetic diversity, broad host range, and ongoing circulation in wild and domestic birds worldwide. As of 29 Nov. 2011, the World Health Organization (WHO) has reported 571 laboratory-confirmed cases of human A/H5N1 infections, resulting in 335 deaths (http://www.who.int/csr/disease/avian_influenza/country/cases_table_2011_01_20/en/index.html). The high observed mortality is a typical feature of this human disease [1]. During the spring of 2009, the emerging swine-origin H1N1 influenza viruses (S-OIVs) are being detected in almost all countries in the world, and their global spread would undoubtedly result in a considerable number of infected individuals [2], [3]. Importantly, a great concern exists that the reassortants between avian H5N1 and influenza A (H1N1), seasonal viruses or changing receptor binding specificity of H5 might be of great impact to human health, once it acquires the capability of human-to-human transmission [4]. Moreover, in the event of a new influenza virus, we cannot predict the strain that will cause the pandemic.

To date, vaccines remain the cornerstone of influenza control. Outbreaks and the pandemic potential of H5N1 viruses have led to stockpiling of H5N1 pre-pandemic inactivated vaccines for human use in many countries. In the face of a highly pathogenic avian influenza (HPAI) H5N1 influenza virus, an update of current and completed vaccine clinical trials can be found on the WHO website (http://www.who.int/vaccine_research/diseases/influenza/flu_trials_tables/en/index.html). The stockpiling of a panel of vaccines with hemagglutinin (HA) antigenic variations, including A/Vietnam/1203/2004(VN), A/Vietnam/1194/2004(VN), A/Indonesia/05/2005(ID), and A/Anhui/1/2005(AH) vaccine viruses, were recommended by the WHO for vaccine development [5]. The H5N1 influenza viruses are currently divisible into clades (0 to 9) on the basis of phylogenetic analysis of their hemagglutinin (HA) genes. The viruses circulating and characterised from 16 February 2011 to 19 September 2011 belonged to the following clades, *Clade 1.1* (previously part of clade 1), *Clade 2.2.1*, *Clade 2.2.1.1* (previously part of clade 2.2.1), *Clade 2.2.2* (previously part of clade 2.2), *Clade 2.3.2.1* (previously part of clade 2.3.2), *Clade 2.3.4*, *Clade 2.3.4.2* (previously part of 2.3.4) [5]. Taken together, most currently circulating H5N1 strains that have infected humans still belong to four serologically distinct antigenic groups (clades 1, 2.1, 2.2, and 2.3.4) [6]. Previous work demonstrated that the multiple-clade vaccine with MF59 adjuvant increased clade-specific and cross-clade antibody responses against lethal challenge with clade 1 and 2 viruses [7]. Although clade 0 was the least frequently seen, during the summer and early fall of 1996, an outbreak of disease with 40% morbidity occurred on a goose farm in Guangdong province, China. The pathogen was isolated and termed A/Goose/Guangdong/1/96(Gs/Gd/1/96) in clade 0. This virus was transmitted to humans and caused deaths [8], [9]. Here, it was unknown if the multiple-clade vaccine based on clade 1 and 2 could provide enough protection against lethal challenge to other clade viruses, such as clade 0, which caused outbreaks in poultry in Hong Kong and was transmitted to humans and caused deaths.

In the present study, we prepared three single H5N1 vaccines, intranasally (i.n.) immunized mice with each vaccine or a trivalent H5N1 influenza split vaccine including clade 1, 2.1 and 2.3.4 viruses of stockpile vaccines with an oil-in-water emulsion adjuvant SP01, and then challenged with heterologus HPAI virus A/OT/SZ/097/03 virus (clade 0) isolated from an ostrich to investigate the immune responses, cross reactivity and a broader cross-protection efficacy in a mouse model.

## Results

### Comparison of the functional efficacy of vaccine groups by hemagglutination inhibition (HI) assays in serum from immunized mice

**Figure 1:**
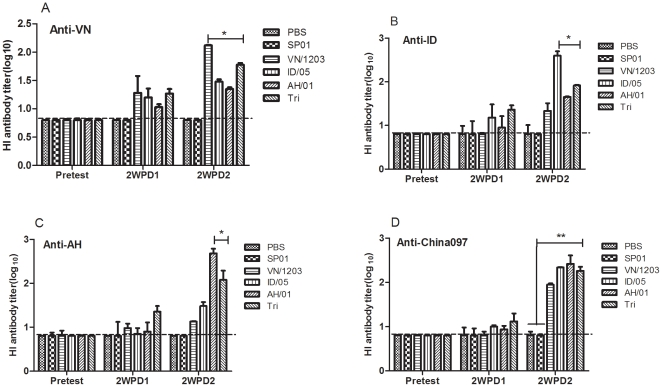
HAI titers of mice immunized with single or trivalent H5N1 influenza split vaccines. Mice were immunized i.n. with single H5N1 influenza split vaccines (3 µg HA per dose) and a trivalent vaccine that contained 1 µg HA per dose of each single vaccine in combination with adjuvant on day 0 and 14, and bled on day 28. Four HA units of VN/1203 (Fig. 1A), ID/05 (Fig. 1B), AH/01 (Fig. 1C), and China097 (Fig. 1D) viral antigen were used. Results are the geometric mean titers of positive sera (HI titer >10). The values are means ± SEM from six mice. * p<0.05 and ** p<0.01. The dashed horizontal line indicates the lower limit of detection.

Sera collected at pretest and 14 days after the first and second doses of vaccine were assayed for the presence of H5N1 influenza-specific antibodies using a HI assay. As shown in Figure 1, at 14 days after the last immunization, the HI titers of the hyper-immune sera from mice immunized with VN/1203(Clade 1), ID/05(Clade 2.1), and AH/01 (Clade 2.3.4) against homologous viruses, reached 1∶125, 1∶400, and 1∶480, respectively. Whereas, the trivalent vaccine elicited humoral immune responses with HI titers reaching 1∶90 against VN/1203, 1∶220 against ID/05, 1∶260 against AH/01, and 1∶185 against China097. Additionally, the results revealed that mice immunized with trivalent vaccine showed a significant rise (P<0.05) of the HI titer against VN/1203, ID/05, AH/01, respectively. Moreover, a significant differrence (P<0.01) was found in the trivalent vaccine against China097 compared to PBS or oil-in-water emulsion groups on day 28. Also, serum from mice vaccinated with single or trivalent vaccine did not have any significant difference on the HI titer over pre-vaccination on day 14 after the primary immunization.

### Determination of mucosal sIgA antibody levels by enzyme-linked immunosorbent assay (ELISA)

**Figure 2:**
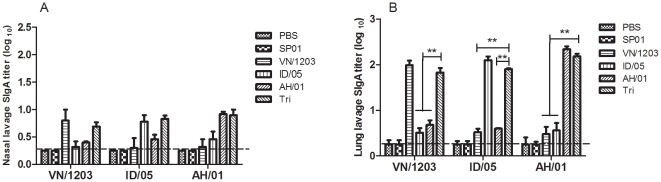
Mucosal antibody response in BALB/c. Secretion of anti-HA IgA antibodies against VN/1203, ID/05, and AH/01 H5N1 inactivated antigen in nasal and lung lavage (dilution 1∶5) from mice immunized i.n. with single H5N1 influenza split vaccines (3 µg HA per dose) and a trivalent vaccine containing 1 µg HA per dose of each single vaccine in combination with adjuvant. The values are means ± SEM from six mice. * p<0.05 and ** p<0.01. The dashed horizontal line indicates the lower limit of detection.

We performed enzyme-linked immunosorbent assays (ELISA) to investigate whether A H5N1 IgA antibodies are present in the mucosal lavage from mice immunized with two doses of a single or trivalent vaccine. In the immunized groups, anti-H5 IgA antibody titers were detected in nasal and lung lavage fluids using ELISA plates coated with purified H5N1 virus antigens 28 days post-immunization. As shown in Figure 2, mice immunized nasally with a single or trivalent vaccine produced mucosal IgA responses, and sIgA antibodies could be detected in nasal and lung lavage fluids, whereas no antibodies were detected in the PBS or oil-in-water emulsion SP01 groups. The results showed that the trivalent vaccine could induce mucosal responses with sIgA titers in lung lavage reaching 1∶66 against VN/1203, 1∶ 80 against ID/05, and 1∶150 against AH/01, which were all lower than with a single vaccine against homologous virus. Moreover, trivalent vaccine against VN/1203, ID/05, or AH/01 significantly enhanced (P<0.01) the HA specific mucosal IgA titer in lung lavage fluids other than against homologous virus coated, whereas sIgA antibody response against the H5N1 virus did not have any significant difference in nasal lavage on day 28. Meanwhile, intramuscular immunization with the H5N1 single or trivalent vaccine failed to induce mucosal antibody responses (data not shown). Thus, an intranasal administration route enhances the efficiency of mucosal antibody production in response to H5N1 vaccination.

### Antibody secreting cell (ASC) response and cytokine assays

**Figure 3:**
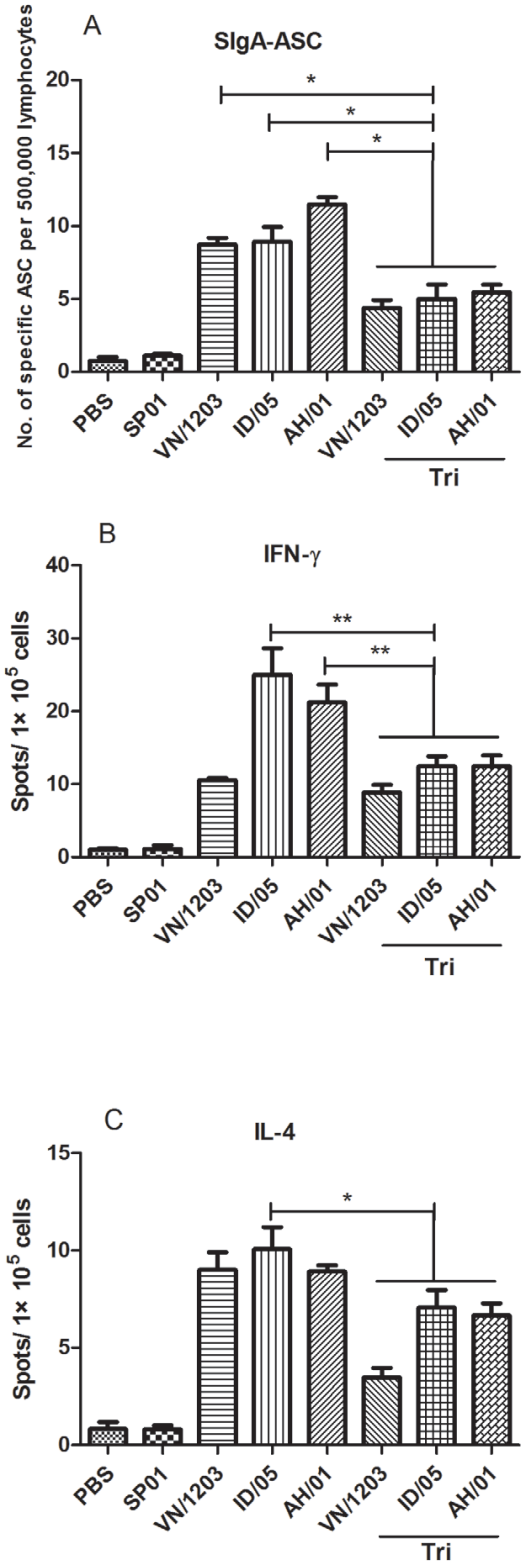
sIgA-ASC, INF-γ, and IL-4 after vaccination as examined by ELISPOT assay. Mice were immunized twice at a 2-week interval with single H5N1 influenza split vaccines (3 µg HA per dose) and a trivalent vaccine that contained 1 µg HA per dose of each single vaccine in combination with adjuvant. On day 14 after the final immunization, pools of three mice for each group were killed and single-cell suspensions were prepared from spleen for the ELISPOT assay. (A) The figure shows antibody-secreting cells (ASCs) per 500,000 lymphocytes. The bars indicate the mean number of specific ASCs per 500,000 lymphocytes ± SD. (B) The INF-γ and IL-4 secreted by spleen lymphocytes were detected using ELISPOT after culture for 40–44 h. The values are means ± SEM from two experiments. * *p*<0.05 and ** *p*<0.01.

No influenza-specific ASCs were detected prior to vaccination. IgA antibody classes dominated the influenza H5N1-specific ASC responses after two doses of the vaccine. As shown in Figure 3A, numbers of ASCs significantly (P<0.05) decreased in mice immunized with trivalent vaccine compared with VN/1203, ID/05, AH/01 single vaccine on day 28 following immunization. Higher numbers of IgA influenza-specific ASCs (ranging from 10 to 90 per 500,000 lymphocytes) were detected on day 28 after the first immunization in spleen lymphocytes of BALB/c mice. With respect to serum IgA responses, little IgA was detected after vaccination (data not shown).

Two weeks after the last immunization, cytokine-producing cell profiles from splenocytes of five mice per group were examined using in vitro stimulation with antigen or RPMI-1640 medium as a negative control. The spot number in each well was calculated on an ELISPOT spot counter. As shown in Figure 3B and 3C, trivalent vaccines induced significant (P<0.01) IFN-γ-producing cells compared to ID/05 or AH/01 single vaccine in immunized BALB/c mice, while only the ID/05 single vaccine group induced significant (P<0.05) specific IL-4-producing cells compared to trivalent vaccines group. In addition, as a stimulus control, splenocytes from each group could not produce IFN-γ or IL-4 while stimulate in vitro with RPMI-1640 medium.

### Protection against challenge with the lethal A/OT/SZ/097/03 (H5N1) virus

**Figure 4:**
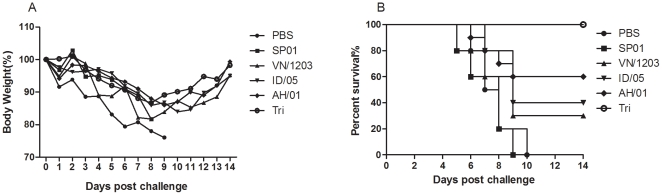
Protective effect of multiple-clade influenza vaccine in mice challenged with A/OT/SZ/097/03. Groups of mice (*n* = 10/group) were immunized i.n. with single H5N1 influenza split vaccines (3 µg HA per dose) and a trivalent vaccine that contained 1 µg HA per dose of each single vaccine in combination with adjuvant and challenged i.n. with 50LD_50_ OT/SZ/097/03 virus suspension. Mice were monitored for weight change (A) and survival rates (B) throughout a 14-day observation period.

**Figure 5:**
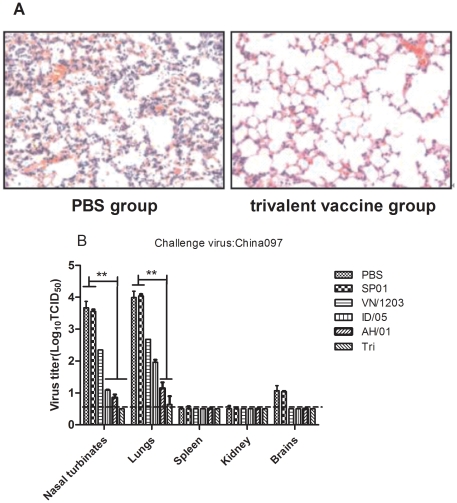
Histopathological changes in lungs and virus titers of infected mice. Mice (*n* = 6/group) immunized twice i.n. with single H5N1 influenza split vaccines (3 µg HA per dose) and a trivalent vaccine that contained 1 µg HA per dose of each single vaccine in combination with adjuvant were infected with 50LD_50_ OT/SZ/097/03 virus 2 weeks after the second immunization. (A) Histopathological changes in the lungs on 4 day post challenge. The figure indicates the representative images of histopathological damage from H & E-stained lungs of 5 mice in PBS group and trivalent vaccine group. (magnification, 100×). (B) On day 4 after virus inoculation, mice were killed and the nasal turbinates, lungs, spleen, kidney, and brains were collected. Virus titers in tissue homogenates were determined by TCID_50_ assay using MDCK cells. The values are means ± SEM from six mice. * p<0.05 and ** p<0.01. The dashed horizontal line indicates the lower limit of detection.

The protective characteristics of standard immunization were tested in an in vivo experiment. We concentrated on the possibility that these single and trivalent vaccines could induce cross protection against the lethal A/OT/SZ/097/03 (H5N1) virus (clade 0). The challenge was made 2 weeks after the last immunization using a lethal clade 0 influenza virus and viral progression was monitored for 14 days. To determined whether intranasal trivalent H5N1 vaccination could elicit protection against lethal cross-infection, immunized mice (*n* = 10/group) were challenged i.n. with a 50LD_50_ (LD_50_ is the dose that causes the death of 50% of test animals) OT/SZ/097/03 H5N1 strain 2 weeks after the boost immunization, and percent survival was recorded. Varying decreases in body weight of the different groups indicated the progress of the infection. In Figure 4A, vaccinated mice were also resistant to lethal challenges with the HPAI H5N1 virus strains, whereas the control animals that received PBS or oil-in-water emulsion adjuvant alone showed clinical signs of severe disease and significant body weight loss starting on day 2 after virus inoculation; they died or reached the humane euthanasia endpoint 7–10 days after challenge. In contrast, all mice immunized with trivalent vaccine remained alive at 14 days after the challenge. Only a transient body weight loss was noted on day 8, but the animals recovered completely during the following week. As shown in Figure 4B, the mortality figures show that the trivalent vaccine provided 100% protection against the OT/SZ/097/03 H5N1 strain while VN/1203, ID/05, and AH/01 vaccine groups provided 30%, 40%, and 60% protection, respectively. Of note, on 4 day post challenge, the group immunized with PBS or oil-in-water emulsion adjuvant SP01 alone showed marked damage to the lung parenchyma, strong lymphocyte infiltration, and slight hemorrhages. A considerably better situation was found in mice immunized using the trivalent vaccine with oil-in-water emulsion adjuvant SP01 (Fig. 5A).

### Replication of the OT/SZ/097/03 H5N1 strain in immunized mice

We investigated whether intranasal immunization with the VN/1203, ID/05, AH/01, and trivalent vaccines could prevent local replication of the OT/SZ/097/03 H5N1 virus in the respiratory tract and systemic spread to the internal organs and brain tissue. Mice (*n* = 6/group) vaccinated twice with VN/1203, ID/05, AH/01, or trivalent vaccines were challenged with the 50LD_50_ OT/SZ/097/03 H5N1 strain, and animals were euthanized on day 4 post challenge. Nasal turbinates, lungs, spleen, kidney and brains were collected to quantify the presence of the *wt* OT/SZ/097/03 virus. Viral titers in nasal turbinates and lungs were reduced significantly (P<0.01) in the group vaccinated with the trivalent vaccine combined with oil-in-water emulsion adjuvant SP01 as compared to the groups administered PBS or adjuvant alone. Moreover, ID/05 and AH/01 vaccine groups had differing degrees of reduction (P<0.01) in nasal turbinates and lungs as compared to placebo. In addition, viral titers in the brain of immunized mice were undetectable in that of all vaccine groups. Meanwhile, no *wt* virus could be detected in the spleen and kidney of immunized mice and control animals (Fig. 5B). Thus, our results show that immunization with VN/1203, ID/05, AH/01, or trivalent vaccines decreases viral titers in the lungs and prevents systemic spread of the OT/SZ/097/03 H5N1 virus in mice.

## Discussion

The increased incidence of human infection with HPAI H5N1 viruses and associated high mortality rates has served as an alarm for the global community. The problem not only concerns the efficacy of the licensed types of influenza vaccines, but also the time and capacity of the world industry to produce sufficient doses of any influenza vaccine for stockpiling. New influenza vaccinations strategies are urgently needed due to uncertainties regarding drift and shift in HPAI viral strains. For, at least 6 months are likely to elapse in face of an influenza pandemic and the initial available of a strain-specific vaccine. An efficient pandemic stockpile vaccine should be capable of inducing cross protection against hypothetical viruses that have not yet spread in the human population. A few results showed after receiving a cross-reactive H5N1 influenza vaccine, individuals may require some immunity to protect from lethal challenge of other clade HPAI H5N1 virus. And after immunizing with a cross-reactive H5N1 influenza vaccine, individuals may require only a single dose of strain-specific pandemic vaccine [7], [10], [11].

The HA sequences of avian H5N1 viruses have diverged into ten distinct phylogenetic clades (genetic groups). Genetic divergence into clades also correlated with antigenic drift and often antibodies generated against one clade do not efficiently inhibit or neutralize a virus from another clade. Some clades have evolved further into second and third class clades (subclades). Most currently circulating H5N1 strains that have infected humans belong to four serologically distinct antigenic groups (clades 1, 2.1, 2.2, and 2.3.4) [10]. However, in 1997, the HPAI H5N1 virus was transmitted directly from chickens to humans and killed 6 of 18 infected people in Hong Kong [12]. Between 2003 and 2005, outbreaks of the H5N1 virus occurred in poultry in East Asian countries, and once again transmission of infection from birds to humans resulted in fatal disease [13]. Influenza *wt* H5N1 virus A/OT/SZ/097/03 was isolated from an ostrich with fatal influenza A H5N1 disease on a farm in 2004 in China. Depending on sequence analysis and cladogram trees, A/OT/SZ/097/03 belongs to Clade 0. In our previous studies, data on antigenic and genetic characteristics of A/OT/SZ/097/03 were reported [14], [15]. According to sequence analyses and cladogram trees, remote antigenic distance exist between VN/1203, ID/05, AH/01 virus and A/OT/SZ/097/03. In this study, we produced influenza H5N1 split vaccines with PR8/H5N1 6∶2 reassortants from isolates of different regions based on the three virus strains of stockpile vaccines, A/Vietnam/1203/2004(Clade 1), A/Indonesia/05/2005(Clade 2.1), and A/Anhui/1/2005(Clade 2.3.4). A mouse vaccination/challenge model was used to assess if this composition with adjuvant could provide cross protection against a lethal challenge with heterologous viruses of A/OT/SZ/097/03(clade 0). Here, we compare the cross-protection efficacy of single, multiple-clade H5N1 influenza vaccines given at 3 µg to a trivalent vaccine given at 1 µg of each virus. Following two immunizations, mice were challenged with a wild type H5N1 virus. Immunological parameters (sIgA ELISA antibody; IFN-γ and IL-4 producing spleen cells) were followed. The key issue is indeed the immunological parameters correlate with protection. Of note, the single vaccines elicited stronger host immune responses to the homologous antigens but less immune response to the heterologous antigens than the trivalent vaccine (Figures 1 and 3), in spite of that the latter contained the same antigens as the former. While the single vaccines also provided less protection against wild type influenza virus A/OT/SZ/097/03 challenge than trivalent vaccine (Figure 5). These results revealed that different antigens of trivalent influenza vaccine could probably overlap in immunogenicity and cross protection and different antigens dose are uncertain equal. Thus, the trivalent vaccine based on clade 1 and 2 was giving rise to a broadly cross-reactive vaccine to potential prevention use ahead of the strain-specific pandemic influenza vaccine in the event of an HPAI H5N1 influenza outbreak.

Cholera toxin (CT) and *E. coli* heat-labile enterotoxin are known potent mucosal vaccine adjuvants and have been used in non-clinical experimental systems. However, their clinical application as nasal adjuvants had to be discontinued because of side effects like Bell's palsy [16]. Therefore, one of the major challenges in adjuvant research is to gain potency while minimizing toxicity. MF59, the first oil-in-water emulsion licensed as an adjuvant for human use, can enhance vaccine immune responses through multiple mechanisms [17], [18]. It is worthy of note that the addition of MF59 adjuvant induced a stronger cross-reactive immunity as compared to non-adjuvanted multiple-clade vaccines [7]. A trivalent MF59-adjuvanted seasonal influenza vaccine has shown to induce significantly higher immune responses to influenza vaccination in the elderly, compared with non-adjuvanted vaccines, and to provide cross-reactive immunity against divergent influenza strains. Similar results have been generated with a MF59-adjuvanted H5N1 pre-pandemic vaccine, which showed higher and broader immunogenicity compared with non-adjuvanted pre-pandemic vaccines [7], [18], [19]. In contrast, we prepared the oil-in-water emulsion adjuvant SP01 and found that mice immunized intranasally with SP01 did not exhibit acute toxicity; i.e., no cytokine-induced mortality, no obvious weight loss, no abnormal behavior and no histopathological changes. In addition, use of SP01 as a nasal adjuvant was safe and effective for inducing remarkably systemic IgG and nasal sIgA Ab responses compared with vaccine antigen alone[20]. Based on above results the single clade and multiple-clade vaccines were tested just with oil-in-water emulsion adjuvant SP01 to investigate this composition vaccine to produce cross protection against heterologous virus challenge.

For development of mucosal vaccines both systemic and mucosal immunity are important. For this reason, a suitable approach would be to develop broad-spectrum virus strains as a backbone; however, currently available single stockpiling influenza virus vaccines are known to be insufficient when used for mucosal immunization and the induction of cross protection [21], [22].Effective H5N1 pandemic preparedness will require vaccines that provide broad cross-protection and are readily available to large groups of the population. However, intranasal delivery of the trivalent vaccine could elicit a broader immune response than the same vaccine delivered intramuscularly (data not shown). Consistent with our previous studies, cellular responses, in particular, were reduced in the pulmonary mucosa of mice vaccinated i.m. as compared to i.n. vaccinated mice. The ability to elicit mucosal immune responses in the respiratory tract, including the lungs, is a desirable characteristic for an influenza vaccine [11].

Naive T-helper cells can differentiate into one of two subsets, known as Th1 and Th2, depending on the context of the initial stimulation they receive [23]. Th1 cells, which secrete IL-2, IFN-γ, and TNF-α, develop in the presence of IL-12. Th1 CD4^+^ T-cells activate CD8^+^ T-cells and prime the cellular immune response. IFN-γ plays a crucial role in the clearance of influenza virus infection via its ability to activate antibacterial functions of infected macrophages. To investigate which subtype of cells is predominant in mice, we examined the amount of IFN-γ- and IL-4-producing cells using an ELISPOT assay. The results show that VN/1203, ID/05, AH/01, and trivalent vaccines could produce more IFN-γ-producing cells than IL-4-producing cells (Fig. 3). Influenza vaccination seems predisposed to generate responses with a strong bias toward TH1-type cellular immune responses because IFN-γ is mainly secreted by Th1 and CD8^+^ T-cells. Our results indicate the importance of Th1-type cellular immune responses in the clearance of influenza viruses from an infected host.

Although some companies, like Baxter, do use inactivated wt H5 viruses as vaccine, the production of H5N1 influenza vaccines from a *wt* virus is generally not acceptable due to the attendant risks and limited application. At present, several technologies are under development for the production of H5N1 pandemic vaccines that have shown promising clinical results. These include vector vaccines, DNA vaccines, adjuvanted vaccines, ancestral sequence reconstruction method and reverse genetics technology [24]–[27]. Using reverse genetics, clinical trials of candidate H5N1 vaccines have been either initiated or completed. These include rg-A/Vietnam/1203/2004(VN), rg-A/Indonesia/05/2005(ID), and rg-A/Anhui/1/2005(AH) [24], [25], [28].

In conclusion, trivalent vaccines containing VN/1203, ID/05, and AH/01 could elicit high immune responses, which provide broader cross protection against lethal heterologus influenza virus infection. The multiple-clade adjuvanted vaccine could be useful in allowing timely initiation of vaccination against unknown pandemic virus. Our findings also suggest that the multiple-clade vaccination strategy appeared, at least in mice, to work successfully for influenza infection and we propose the preferential stockpiling of the pre-pandemic trivalent vaccines. Potential problems, including safety considerations and reactions among vaccine antigens, require further studies. Additionally, our findings prompt further investigations of the effect of the multiple-clade H5N1 influenza vaccines in ferrets, monkeys and man.

## Materials and Methods

### Animals

We purchased 6- to 8-week-old female BALB/c mice from the Laboratory Animal Center of the Academy of Military Medical Sciences (AMMS), Beijing, China. Mice were kept under specific-pathogen-free (SPF) conditions approved by the Institutional Animal Care and Use Committee of the AMMS (ID: SYXK 2007-005). All operations with the wild-type virus A/OT/SZ/097/03 was performed in biosafety level 3 animal facilities.

### Viruses

Influenza vaccine strains rg-A/Vietnam/1203/2004, rg-A/Indonesia/05/2005, and rg-A/Anhui/01/2005 (abbreviated VN/1203, ID/05, and AH/01, respectively) were derived from a recombinant avirulent avian virus containing modified HA and neuraminidase NA genes of the wt H5N1 virus in the background A/Puerto Rico/8/34. These vaccine strains were supplied by the UK NIBSC and US Centers for Disease Control and Prevention. The strain of HPAI H5N1 *wt* virus A/OT/SZ/097/03 (H5 HA clade 0) (abbreviated China097), which was used as a challenge in this study, was isolated and identified from fecal samples of a dead ostrich on a farm at Yangzhou University in Jiangsu Province [14]. These viruses were propagated in 10-day-old embryonated chicken eggs for 2 days at 35°C, and then stored at −80°C. Virus titers were quantified by TCID_50_ assay using MDCK cells (ATCC CCL-34) [29].

### Preparation of vaccine

Influenza vaccine strains rg-A/Vietnam/1203/2004, rg-A/Indonesia/05/2005, and rg-A/Anhui/1/2005 are pandemic vaccine strains recommended for use in vaccine development. The vaccine was formulated and produced by Hualan Vaccine Inc. (Henan, China) as influenza H5N1 split vaccines, with or without adjuvant, and supplied in 0.5-ml pre-filled single-dose syringes. Briefly, the virus vaccine was propagated in the allantoic cavity of 10-day-old SPF embryonating chicken eggs at 35°C. Allantoic fluids were harvested 48 h after inoculation. Virus titers were determined using HA assays. The vaccine was generated according to standard techniques. The seed virus was grown to a high titer in eggs and the virions were purified by centrifugation, inactivated with formalin, and filtered to remove bacteria. Influenza H5N1 split vaccines were produced on a pilot scale under Good Manufacturing Practices. The HA protein content of the vaccines was determined by the single radial immunodiffusion (SRID) method [30]. Oil-in-water emulsion adjuvant SP01 containing squalene, polyether and castor oil was added by mixing 1∶1 v/v with the vaccine.

### Immunization and sample collection

Twenty-two mice for each experimental group were anesthetized by intraperitoneal injection of 0.2 ml 2,2,2-tribromoethanol in *tert*-amyl alcohol (Avertin; Sigma-Aldrich, St. Louis, MO) and vaccinated i.n. with 3 µg influenza split vaccine (based on HA content) in 20 µl phosphate-buffered saline (PBS) with an adjuvant. Single vaccines (3 µg HA per dose) included VN/1203, AH/01, and ID/05. The trivalent vaccine contained 1 µg HA per dose of each single vaccine. SP01 adjuvants were used in both primary and secondary vaccination [20]. Fourteen days post-primary vaccination, mice were boosted with a second dose of 3 µg H5N1 influenza split vaccine by the i.n. route with an adjuvant. Six mice were killed on day 28 and blood was collected. Meanwhile, lung, nasal cavity, and vaginal lavage were collected for measurement of mucosal IgA and IgG titers as described previously [31].

### Hemagglutination inhibition (HI)

Serum samples were taken pretest, 2 weeks after the first vaccination, and 2 weeks after the boost dose. Reference viruses used for serologic testing were reverse genetics strains, and all serological testing was performed in the BSL3+ containment facility. Serum was treated with receptor-destroying enzyme overnight at 37°C, heat-inactivated at 56°C for 45 min, diluted 1∶10 with sterile PBS, and tested by hemagglutination inhibition (HI) assay with 0.5% turkey erythrocytes [32]. The sera from mice administrated PBS were used as negative controls. All assays were performed in triplicate and repeated three times.

### Preparation of splenocytes and ELISPOT assay

Two weeks after the final immunization, the spleens of dead mice were removed under aseptic conditions. Single-cell suspensions were prepared from the spleen, and the red blood cells were lysed with red blood cell lysis buffer. Splenocytes were assayed immediately at 37°C in 5% CO_2_ in RPM-1640 medium supplemented with the appropriate heat-inactivated fetal calf serum. The number of antibody-secreting cells (ASCs) was evaluated in an enzyme-linked immunoSPOT assay (ELISPOT) with the following modifications. ELISPOT plates were coated overnight at 4°C, with 5 µg/ml split virus vaccine. Lymphocytes (1×10^5^ cells) were added in duplicate and incubated overnight. Antibodies (IgG, IgA, and IgM) secreted by the lymphocytes were detected by incubation with biotin-labeled goat anti-mouse class-specific antibodies for 2 h at room temperature. Following development, the number of spots was counted using an ImmunoSPOT series 3 automatic ELISPOT Reader.

To measure the amount of cytokine-producing cells, after 24 h of stimulation in vitro with 7.5 µg/ml virus antigen, spleen cells were tested for the presence of IFN-γ and IL-4 by ELISPOT assay using a BD TM mouse IFN-γ and IL-4 ELISPOT set [33]. The spot number corresponding to the IFN-γ- and IL-4-producing cells was calculated on an ELISPOT spot counter. All assays were performed in triplicate and repeated three times.

### Protection experiments

Ten mice for each experimental group were anaesthetized with 2,2,2-tribromoethanol in *tert*-amyl alcohol (Avertin; Sigma-Aldrich, St. Louis, MO) and immunized intranasally with 3 µg HA amount of vaccine with SP01 adjuvant. Two weeks later, they were re-immunized in the same manner. Protection experiments were performed by challenging mice with a nasal administration of 50LD_50_ A/OT/SZ/097/03 H5N1 virus 2 weeks after the last immunization. Mice were followed 14 days after the infection, and survival and weight loss were monitored. Lung tissues were collected from euthanized mice on 4 day post-challenge for further virological testing and histopathological analysis.

### Viral titers

Immunized mice were challenged as described above and killed on day 4 after virus inoculation. Nasal turbinates, lungs, spleen, kidney and brains were harvested and homogenized in MEM medium containing antibiotics to make a 10% w/v tissue homogenates on day 4 post-infection (p.i.). Clarified tissue homogenates by low-speed centrifugation were titrated in 24- and 96-well culture plates containing MDCK cells, and titers were expressed as log_10_TCID_50_/gram tissue [29].

### Histopathological analysis

The lung tissues of challenged mice were immediately fixed in 10% neutral buffered formalin and embedded in paraffin wax. Sections were made at 4–6 µm thickness and mounted on slides. Histopathological changes were examined by H & E staining and observed under light microscopy.

### Statistical analyses

All data are shown as means ± S.E.M. Measurements at single timepoints were analyzed by an ANOVA, and if they demonstrated significance, they were further analyzed by a two-tailed t-test. All statistical tests were conducted using GraphPad Prism 5 software. p<0.05 indicates statistical significance.
